# The safety and efficacy of fingolimod: Real-world data from a long-term, non-interventional study on the treatment of RRMS patients spanning up to 5 years from Hungary

**DOI:** 10.1371/journal.pone.0267346

**Published:** 2022-04-22

**Authors:** Tamás Biernacki, Dániel Sandi, Judit Füvesi, Zsanett Fricska-Nagy, Tamás Zsigmond Kincses, Péter Ács, Csilla Rózsa, Enikő Dobos, Botond Cseh, László Horváth, Zsuzsanna Nagy, Attila Csányi, Krisztina Kovács, Tünde Csépány, László Vécsei, Krisztina Bencsik, on the behalf of the study investigators

**Affiliations:** 1 Department of Neurology, Faculty of General Medicine, Albert Szent-Györgyi Clinical Centre, University of Szeged, Szeged, Hungary; 2 Department of Neurology, Faculty of General Medicine, University of Pécs, Pécs, Hungary; 3 Jahn Ferenc South-Pest Hospital and Clinic, Budapest, Hungary; 4 Szent Imre Hospital, Budapest, Hungary; 5 Borsod-Abaúj-Zemplén County Hospital, Miskolc, Hungary; 6 Szent Rafael Zala County Hospital, Zalaegerszeg, Hungary; 7 Petz Aladár County Teaching Hospital, Győr, Hungary; 8 Péterfy Sándor str. Hospital and Clinic, Budapest, Hungary; 9 Department of Neurology, Faculty of General Medicine, University of Debrecen, Deberecen, Hungary; Universita degli Studi di Napoli Federico II, ITALY

## Abstract

**Background:**

Fingolimod was approved and reimbursed by the healthcare provider in Hungary for the treatment of highly active relapsing-remitting multiple sclerosis (RRMS) in 2012. The present study aimed to assess the effectiveness, safety profile, and persistence to fingolimod in a real-life setting in Hungary in RRMS patients who were either therapy naïve before enrollment or have changed to fingolimod from another disease-modifying therapy (DMT) for any reason.

**Methods:**

This cross-sectional, observational study with prospective data collection was performed nationwide at 21 sites across Hungary. To avoid selection bias, sites were asked to document eligible patients in consecutive chronological order. Demographic, clinical, safety and efficacy data were analysed for up to 5 years from 570 consenting adult patients with RRMS who had received treatment with fingolimod for at least one year.

**Results:**

69.6% of patients remained free from relapses for the whole study duration; in the first year, 85.1% of patients did not experience a relapse, which rose to 94.6% seen in the 5th year. Compared to baseline at study end, 28.2% had higher, and 9.1% had lower, meanwhile, 62.7% of the patients had stable EDSS scores. Overall, the annualized relapse rate decreased from 0.804 observed at baseline to 0.185, 0.149, 0.122, 0.091, and 0.097 (77.0%, 82.1%, 85.2%, 89.7%, and 89.0% relative reduction, respectively) after 1, 2, 3, 4, and 5 years of treatment. The greatest reduction rate was seen in the group of therapy naïve patients. Treatment persistence on fingolimod after 60 months was 73.4%.

**Conclusion:**

In this nationwide Hungarian cohort, most patients under fingolimod treatment were free from relapses and disability progression. In addition, fingolimod has proven to be a well-tolerated DMT that has sustained its manageable safety profile, high efficacy, and positive benefit/risk ratio for up to 5 years in a real-life setting.

## Introduction

Multiple sclerosis (MS) is the most common autoimmune, inflammatory demyelinating, and neurodegenerative disease of the central nervous system. Even though it is considered a rare disease, it affects more than 2 million people worldwide. Also, its incidence, especially in women, has been reported to show a constant increase in the past decades [1]. MS is also the second most common cause of permanent disability among young adults, preceded only by traumatic injury [2–4]. The most frequent form of MS is the relapsing-remitting subtype (RRMS), where high disease activity indicated by recurrent relapses and various MRI parameters, especially in the initial phase of the disease, have been found to predict future disease activity and have been linked to poor long-term prognosis [5–7]. At the beginning of the disease, inflammatory events dominate; however, the extent of their effect on the rate of the concomitantly ongoing (and later on overtaking) neurodegenerative processes is not fully established yet. Irreversible axonal injury and transition into a progressive disease course have been suggested to occur approximately at an expanded disability status scale (EDSS) [8] score of 4 [9]. The so-called “therapeutic window” to stabilise a patient’s illness and prevent future, permanent disability and transition into the progressive phase seem to be before this point [10]. Therefore, commencing a treatment tailored to a patient’s disease activity as early as possible is crucial. Fingolimod by modulating the sphingosine-1-phosphate type 1 receptor (S1P1) renders both B and T cells insensitive to signals from secondary lymphoid tissues [11], preventing them from leaving the lymph nodes, pushes macrophages toward an anti-inflammatory M2 phenotype [12] and stimulates the repair process of glial cells and glial precursor cells following injury [13].

Fingolimod was approved in 2010 by the Food and Drug Administration (FDA) in the U.S. and early 2011 by the European Medicines Agency (EMA) in Europe as the first oral disease-modifying therapy (DMT) initially for the treatment of adult patients with RRMS [14]. Later, in 2018 it was approved for the treatment of adolescent RRMS patients as well. In Hungary, fingolimod was introduced in 2012 and received full reimbursement by the healthcare provider in 2014, to be used as either first or second-line drug for the treatment of RRMS patients. Every new drug is mandatorily evaluated in randomised controlled trials (RCTs) on carefully selected, homogenous study populations under rigorous experimental conditions to generate data for regulatory approval. The beneficial effects of fingolimod on disability progression, annual relapse rate (ARR), radiological activity and brain volume loss were proven in three pivotal phase III trials [15–17]. The extensions of these trials have shown benefits and sustained efficacy in the long term, but due to the nature of clinical trials, their value is limited, however. A major pitfall of even the most precisely conducted RCTs is their limited validity, because in many cases the measured outcomes may not be generalisable to the everyday clinical practice [18–20]. In contrast to RCTs, the inclusion criteria of real-world studies are usually not as rigorous; thus, they allow for the observation of a more heterogeneous and larger study population, which more adequately reflects the clinical environment in which the drug is going to be used on the long run. Real-life studies can furthermore assess outcomes and measure the effectiveness of treatment over a more extended period. Meanwhile, they can identify rare side effects of the medication administered, which remained unearthed in the initial RCTs. Also, they give complementary data to traditional clinical trial outcome measures by providing information regarding patients’ persistence to therapy. Real-life studies also frequently employ patient-reported outcomes (PROs), which can provide a glimpse into the everyday life of the patients and reveal the influence of the administered treatment on it, aspects which otherwise would be left unrecognised [21–24]. In recent years several long-term observational studies assessed the sustained efficacy of fingolimod, but excluding very few exceptions, many of these trials were either conducted on the patients participating in the pivotal trials (extension studies and various post hoc analyses), or have included only a relatively small amount of patients, and/or followed the patients only for a brief period [25–34].

Therefore, we aimed to create a study using loose enough inclusion criteria with a long-term follow-up period to ensure the participation of the largest number of patients possible while employing a vigorous follow-up schedule resembling that seen in RCTs. The objectives of our study were to evaluate the patients’ adherence to treatment, to assess the long-term efficacy of fingolimod after at least 12 months of treatment up to 5 years, to characterise the demographic and clinical profile of patients receiving treatment and also to assess the safety profile of fingolimod in a real-life setting in Hungary.

## Materials and methods

### Study design

Ours was a multicenter, prospective, non-interventional, observational, nationwide long-term study. Patients were enrolled from 21 MS centres across Hungary. Inclusion criteria were: written informed consent to the study; participants had to be at least 18 years old; diagnosis of RRMS always in accordance with the latest revised McDonald criteria (the 2010 revision was used for patients enrolled between 2014–2017, and the 2017 revision was used for patients recruited after 2017); to have been prescribed fingolimod 0.5 mg by their physician as part of routine clinical practice. Exclusion criteria were: any contraindication in the European fingolimod summary of product characteristics (SmPC); concomitant major depression; simultaneous participation in another study; the presence of cognitive impairment severe enough to not be able to fill out the questionnaires used in the study without assistance.

The primary endpoint was the change in the annualised relapse rate. Secondary endpoints included the change of EDSS score compared to baseline, accumulation of 6-month confirmed disability, treatment persistence (defined as the proportion of patients who, at a given time, continued to receive fingolimod, there were no known patients included in the study who skipped doses), the change of health-related quality of life (HRQoL) based on patient-reported outcomes (we used the validated Hungarian version of the MSQOL-54 questionnaire [35]) compared to baseline, changes on the Clinical Global Impression Questionnaire (CGI) compared to baseline, and the safety profile of long-term fingolimod treatment. For the detailed follow-up protocol employed, see the S1 Table.

The maximum length of the observation was 5 years; the observation period spanned from the 1st of October 2012 to the 28th of June 2019. Recruitment of new patients started on the 1st of October 2012 and stopped on the 31st of December 2018. Procedures were in concordance with the guidelines established in the Declaration of Helsinki and performed following Good Clinical Practice guidelines. The study was conducted in accordance with the Declaration of Helsinki and approved by the Hungarian Medical Research Council (reference number: 28651/2011/EKU, IV/3910-3/2021/EKU). At enrollment, written consent was obtained from all participants.

Initially, a total of 720 patients (S2 Table) had been enrolled in the study, patients who had received at least one dose of fingolimod, and thus constituted the safety population (SP). During the observation period, 150 patients had dropped out; 143 patients did not complete 12 months of follow-up until study closure, meanwhile 7 patients had withdrawn their consent to participate. In total, 570 patients (intention to treat—ITT population) have completed at least 1 year of treatment; 478, 370, 262, and 184 patients were followed for 2, 3, 4 and 5 years, respectively. Except for the safety data (for which the SP was used), all our results are based on the ITT population. For various analyses, patients were stratified into 4 subgroups based on the previous disease-modifying treatments (DMT) they have used just prior to fingolimod: the Naïve group for patients who did not receive any prior DMT, the INJ group for those who were previously treated with an injectable DMT (subcutaneous or intramuscular IFN beta-1a, subcutaneous IFN beta-1b, and subcutaneous glatiramer acetate), patients who switched to fingolimod from natalizumab constituted the NTZ group, the Other group included patients who had been switched to fingolimod from a DMT other than the previously mentioned ones.

### Measures

The socio-demographic and clinical characteristics (MS subtype, age at disease onset, disease duration, EDSS score, prior MS treatments, number of relapses, and ARR before study start) were recorded at enrollment, before treatment initiation. Treatment effectiveness and safety measures were continuously collected during the study. The ARR was assessed in the year prior to commencement of fingolimod treatment and after that in each year during the study.

Clinical outcomes and the outcome of all adverse events (AEs) and serious AEs (SAEs) were always assessed by the given patient’s treating neurologist. The level of disability was determined by using the EDSS scale. Stable EDSS was defined as less than a 1.0 point change if the EDSS score was between 1.0–4.5, less than a 1.5 point increase from an EDSS score of 0, and no change from an EDSS score of at least 5.0 points. Disability progression was defined as an increase of at least 1.5 points from an EDSS score of 0, at least 1 point from an EDSS score of between 1 and 4.5, or at least 0.5 points from an EDSS score greater or equal to 5.0. Improvement was defined as at least a 1.0 point decrease from an EDSS between 1.0–4.5, and at least a 0.5 point decrease if the EDSS was at least 5.0. Disability progression was always confirmed on the patient’s next visit according to routine clinical practice (i.e. once every three months) in the absence of a relapse at the time of assessment.

### Statistical analyses

Data for categorical variables are presented as the number of cases and the proportion of patients in each category. Data are summarised using the mean, 95% confidence interval (CI), and standard deviation (SD) for continuous variables. For proportions of patients, 95% CIs were calculated using the exact (Clopper–Pearson) method. Time to therapy discontinuation, relapse-free and disease progression-free survival was estimated using a Kaplan–Meier approach. Non-parametrical tests were used due to the non-normal distribution. Friedman ANOVA test was used to analyse the multivariable statistics. The Wilcoxon matched-pairs test was used to assess the statistical significance when parts of variables were used. ARR and associated 95% CIs were analysed using a negative binomial distribution model and logarithm of time on study as an offset variable. If patient data were missing or if patients were lost to follow-up, data were taken into consideration up to the point of discontinuation.

## Results

### Study population and baseline characteristics

The ITT population consisted of 178 (31.2%) men and 392 (68.8%) women; their mean age was 39.14 ± 9.79 years (median: 39 years), age at disease onset was 28.7 ± 8.5 years. The mean disease duration for the whole cohort was 10.3 ± 6.7 years. There was a significant difference between the subgroups: patients in the Naïve group were younger (p = 0.045), had a shorter disease course (p = 0.045) and had higher ARR (p = 0.017) than patients in the other subgroups. Meanwhile, patients in the NTZ group were significantly younger than patients in the other subgroups at both the time of disease onset and diagnosis. Also, patients in the NTZ group had the highest (2.97 [±1.61]), and patients in the Naïve group had the lowest (2.27 [±1.15]) EDSS scores (p<0.05 for both comparisons) (Tables 1 and 2).

**Table 1:** Baseline demographic and clinical characteristics for the intention to treat population.

		Whole cohort	INJ	NTZ	Naïve	Other
**Age (mean years, ± SD)**		**39.14 (± 9.79)**	**39.32 (±10.32)**	**39.08 (±9.07)**	**35.83 (±8.45)**	**40.12 (±9.50)**
**Sex (No., %)**	**Male**	**178 (31.2%)**	**75 (27.9%)**	**38 (31.1%)**	**20 (33.9%)**	**43 (36.8%)**
	**Female**	**392 (68.8%)**	**194 (72.1%)**	**84 (68.9%)**	**39 (66.1%)**	**74 (63.2%)**
**Age at first symptom (mean years, ± SD)**		**28.66 (±8.46)**	**28.25 (±8.70)**	**27.41 (±7.78)**	**30.78 (±7.51)**	**29.87 (±8.70)**
**Age at diagnosis (mean years, ± SD)**		**30.46 (±8.81)**	**30.36 (±9.06)**	**28.35 (±7.71)**	**32.19 (±7.75)**	**31.81 (±9.35)**
**Time from diagnosis (mean years, ± SD)**		**8.46 (±6.19)**	**8.84 (±6.27)**	**10.45 (±5.34)**	**3.10 (±5.55)**	**8.11 (±5.64)**
**Time from first symptom (mean years, ± SD)**		**10.27 (±6.67)**	**10.86 (±6.78)**	**11.45 (±6.15)**	**4.80 (±5.63)**	**10.06 (±5.75)**
**EDSS at study baseline (mean points, ± SD)**		**2.67 (±1.52)**	**2.65 (±1.60)**	**2.97 (±1.61)**	**2.27 (±1.15)**	**2.59 (±1.34)**
**EDSS at study baseline (median, min-max)**		**2.5 (0.0–7.0)**	**2.5 (0.0–7.0)**	**3.0 (0.0–6.5)**	**2.0 (1.0–5.0)**	**2.0 (0.0–6.0)**
**Previous smoker (No., %)**	**Yes**	**102 (18.0%)**	**53 (21.3%)**	**17 (15.2%)**	**10 (20.4%)**	**22 (21.6%)**
	**No**	**410 (72.3%)**	**196 (78.7%)**	**95 (84.8%)**	**39 (79.6%)**	**80 (78.4%)**
	**Unknown** ^a^	**55 (9.7%)**	**n/a**	**n/a**	**n/a**	**n/a**
**Current smoker (No., %)**		**82 (80.4%)**	**43 (86.0%)**	**12 (70.6%)**	**7 (70.0%)**	**20 (90.9%)**
**Average No. of previous relapses (mean, ± SD)**		**4.93 (±3.06)**	**4.92 (±2.72)**	**5.87 (±3.50)**	**2.53 (±1.19)**	**5.22 (±3.38)**
**Number of relapses prior study start (No., %)**	**Unknown**	**5 (0.9%)**	**2 (0.1%)**	**3 (2.4%)**	**0 (0.0%)**	**0 (0.0%)**
	**1**	**32 (5.6%)**	**6 (2.2%)**	**5 (4.1%)**	**13 (22.0%)**	**8 (6.8%)**
	**2**	**70 (12.3%)**	**29 (10.8%)**	**10 (8.2%)**	**18 (30.5%)**	**13 (11.1%)**
	**3**	**107 (18.8%)**	**55 (20.5%)**	**16 (13.1%)**	**16 (27.1%)**	**19 (16.2%)**
	**4**	**99 (17.4%)**	**50 (18.6%)**	**19 (15.6%)**	**9 (15.3%)**	**20 (17.1%)**
	**5**	**76 (13.3%)**	**42 (15.6%)**	**16 (13.1%)**	**2 (3.4%)**	**15 (12.8%)**
	**6**	**59 (10.4%)**	**31 (11.5%)**	**13 (10.7%)**	**1 (1.7%)**	**14 (12.0%)**
	**7**	**34 (6.0%)**	**22 (8.2%)**	**9 (7.4%)**	**0 (0.0%)**	**3 (2.6%)**
	**8**	**31 (5.4%)**	**10 (3.7%)**	**12 (9.8%)**	**0 (0.0%)**	**9 (7.7%)**
	**9+**	**57 (10.0%)**	**22 (8.2%)**	**19 (15.6%)**	**0 (0.0%)**	**16 (13.7%)**
**Average time on fingolimod (months)**		**32.43 (±17.32)**	**35.84 (±17.10)**	**38.01 (±16.50)**	**24.42 (±15.70)**	**22.79 (±17.32)**
**No. of previously used DMTs**	**0**	**59 (10.4%)**	**0 (0.0%)**	**0 (0.0%)**	**59 (100%)**	**0 (0.0%)**
	**1**	**260 (45.6%)**	**195 (72.5%)**	**19 (15.6%)**	**0 (0.0%)**	**46 (39.3%)**
	**2**	**181 (31.8%)**	**70 (26.0%)**	**68 (55.7%)**	**0 (0.0%)**	**43 (36.8%)**
	**3**	**59 (10.4%)**	**4 (1.5%)**	**30 (24.6%)**	**0 (0.0%)**	**25 (21.3%)**
	**4**	**8 (1.4%)**	**0 (0.0%)**	**5 (4.1%)**	**0 (0.0%)**	**3 (2.6%)**
**Previously used DMT**	**Dimethyl fumarate**	**53 (9.3%)**	**0 (0.0%)**	**0 (0.0%)**	**0 (0.0%)**	**53 (45.3%)**
	**Glatiramer acetate**	**175 (30.7%)**	**102 (37.9%)**	**49 (40.2%)**	**0 (0.0%)**	**24 (20.5%)**
	**Interferons**	**395 (69.6%)**	**263 (97.8%)**	**81 (66.4%)**	**0 (0.0%)**	**51 (43.6%)**
	**Natalizumab**	**133 (23.3%)**	**0 (0.0%)**	**122 (100.0%)**	**0 (0.0%)**	**11 (9.4%)**
	**Naïve**	**59 (10.4%)**	**0 (0.0%)**	**0 (0.0%)**	**59 (100%)**	**0 (0.0%)**
	**Other** ^b^	**38 (6.6%)**	**0 (0.0%)**	**0 (0.0%)**	**0 (0.0%)**	**38 (100.0%)**
	**Teriflunomide**	**36 (6.3%)**	**0 (0.0%)**	**0 (0.0%)**	**0 (0.0%)**	**36 (30.8%)**

**Notes**: no information was available about three patients (0.5%),

^a^: no data was supplied about three patients regarding the DMT used immediately before fingolimod,

^b^: other therapies included: azathioprine, mitoxantrone, daclizumab, siponimod, and generic interferon derivatives.

**Abbreviations**: DMT, disease-modifying treatment.

**Table 2:** Efficacy of fingolimod stratified by previous DMT used.

			Whole cohort	INJ	NTZ	Naïve	Other	P value (inter groups)
**ARR** **(mean, 95% CI)**	**prior to study start**		**0.80 (0.74–0.86)**	**0.87 (0.77–0.97)**	**0.72 (0.65–0.80)**	**1.04 (0.68–1.41)**	**0.69 (0.61–0.78)**	**0.017**
	**Year 1**		**0.19 (0.14–0.23)**	**0.15 (0.10–0.21)**	**0.29 (0.17–0.40)**	**0.10 (0.02–0.18)**	**0.18 (0.10–0.25)**	**0.008**
	**Year 2**		**0.15 (0.11–0.19)**	**0.14 (0.09–0.19)**	**0.13 (0.06–0.21)**	**0.10 (0.02–0.20)**	**0.12 (0.03–0.21)**	**0.902**
	**Year 3**		**0.12 (0.08–0.17)**	**0.09 (0.05–0.13)**	**0.19 (0.08–0.29)**	**0.16 (0.00–0.33)**	**0.08 (0.00–0.16)**	**0.124**
	**Year 4**		**0.09 (0.04–0.14)**	**0.10 (0.04–0.16)**	**0.13 (0.04–0.22)**	**0.05 (0.00–0.17)**	**0.00 (0.00–0.00)**	**0.481**
	**Year 5**		**0.10 (0.03–0.16)**	**0.06 (0.02–0.11)**	**0.09 (0.00–0.20)**	**0.00 (0.00–0.00)**	**0.00 (0.00–0.00)**	**0.772**
**EDSS Score**	**Baseline**	**mean (± SD)**	**2.67 (±1.52)**	**2.65 (±1.60)**	**2.97 (±1.61)**	**2.27 (±1.15)**	**2.59 (±1.34)**	**0.043**
		**median (min-max)**	**2.5 (0.0–7.0)**	**2.5 (0.0–7.0)**	**3.0 (0.0–6.5)**	**2.0 (1.0–5.0)**	**2.0 (0.0–6.0)**	
	**Year 1**	**mean (± SD)**	**2.66 (±1.66)**	**2.62 (±1.73)**	**3.03 (±1.63)**	**1.88 (±1.20)**	**2.69(±1.60)**	**0.002**
		**median (min-max)**	**2.5 (0.0–8.5)**	**2.5 (0.0–8.5)**	**3.0 (0.0–6.5)**	**1.5 (0.0–5.5)**	**2.5 (0.0–6.5)**	
	**Year 2**	**mean (± SD)**	**2.85 (±1.75)**	**2.76 (±1.75)**	**3.19 (±1.75)**	**1.92 (±1.40)**	**3.12 (±1.74)**	**0.008**
		**median (min-max)**	**2.5 (0.0–7.0)**	**2.5 (0.0–7.0)**	**3.0 (0.0–6.5)**	**1.5 (0.0–5.5)**	**3.0 (0.0–6.5)**	
	**Year 3**	**mean (± SD)**	**2.96 (±1.86)**	**2.95 (±1.85)**	**3.37 (±1.81)**	**1.73 (±1.63)**	**2.82 (±1.82)**	**0.005**
		**median (min-max)**	**2.5 (0.0–7.5)**	**2.5 (0.0–7.5)**	**3.0 (0.0–7.0)**	**1.25 (0.0–6.0)**	**2.0 (0.0–6.5)**	
	**Year 4**	**mean (± SD)**	**3.16 (±1.89)**	**2.88 (±1.88)**	**4.02 (±1.76)**	**2.08 (±2.08)**	**2.23 (±1.06)**	**<0.001**
		**median (min-max)**	**3.0 (0.0–8.5)**	**2.5 (0.0–8.5)**	**4.0 (1.0–7.5)**	**1.5 (0.0–6.0)**	**2.0 (1.0–4.0)**	
	**Year 5**	**mean (± SD)**	**3.32 (±1.92)**	**3.08 (±1.98)**	**3.99 (±1.77)**	**1.88 (±1.44)**	**3.00 (±1.08)**	**0.048**
		**median (min-max)**	**3.5 (0.0–7.5)**	**2.5 (0.0–7.5)**	**4.0 (1.0–6.5)**	**1.25 (1.0–4.0)**	**3.25 (1.5–4.0)**	

**Abbreviations**: ARR, annualised relapse rate; EDSS, expanded disability status scale.

A total of 305 (53.50%) patients had at least one known comorbidity prior to study start, 337 (59.12%) subjects lived with at least one concomitant disease at study closure. The most common acquired diseases were of vascular (hypertension– 117 patients, 20.53%), psychiatric (depression– 57 patients, 10.00%, anxiety—23 patients, 4.14%, depressive disorder– 78 patients, 13.68%) and nervous system (migraine—patients 20, 3.50%) origin. More than half (372, 65.26%) of the patients used at least one medication other than fingolimod to treat a concurrent disease. The most common medications were anxiolytics and antiepileptic drugs (alprazolam– 97, 17.01%, clonazepam– 38, 6.66%), muscle relaxants (baclofen– 86, 15.08%, tizanidine– 32, 5.61%), antidepressants (sertraline– 32, 5.61%, citalopram– 29, 5.08%) and urological drugs (oxybutynin– 32, 5.61%).

In the ITT population, fingolimod was the first treatment choice for MS for 59 patients. The rest had already used another immunomodulatory drug for MS. The most common DMTs prescribed to patients prior to fingolimod were injectables (interferon beta-1a, interferon beta-1b, and glatiramer acetate), with the most frequently used being glatiramer acetate (175 patients, 30.70%) (Table 1). In the INF group, the most common reason for the discontinuation and change to fingolimod was the lack of effectiveness of the previous treatment (53–65%). In the Other group, reasons for the change were mixed (etc. side effects for dimethyl fumarate—71.4%, lack of efficacy for teriflunomide– 66.6%). The main drive for the change in the NTZ group was the increased risk of progressive multifocal leukoencephalopathy (75%).

The vast majority (565, 99.1%) of patients in the ITT population suffered at least one relapse before study start; megadose steroid therapy was necessary for the treatment of 553 (97.0%) patients’ relapses, the mean number of relapses before study start was 4.93 ± 3.01 (median 4.0) (Table 1).

### Effectiveness

#### Disease activity, relapses, and disability

**Fig 1:**
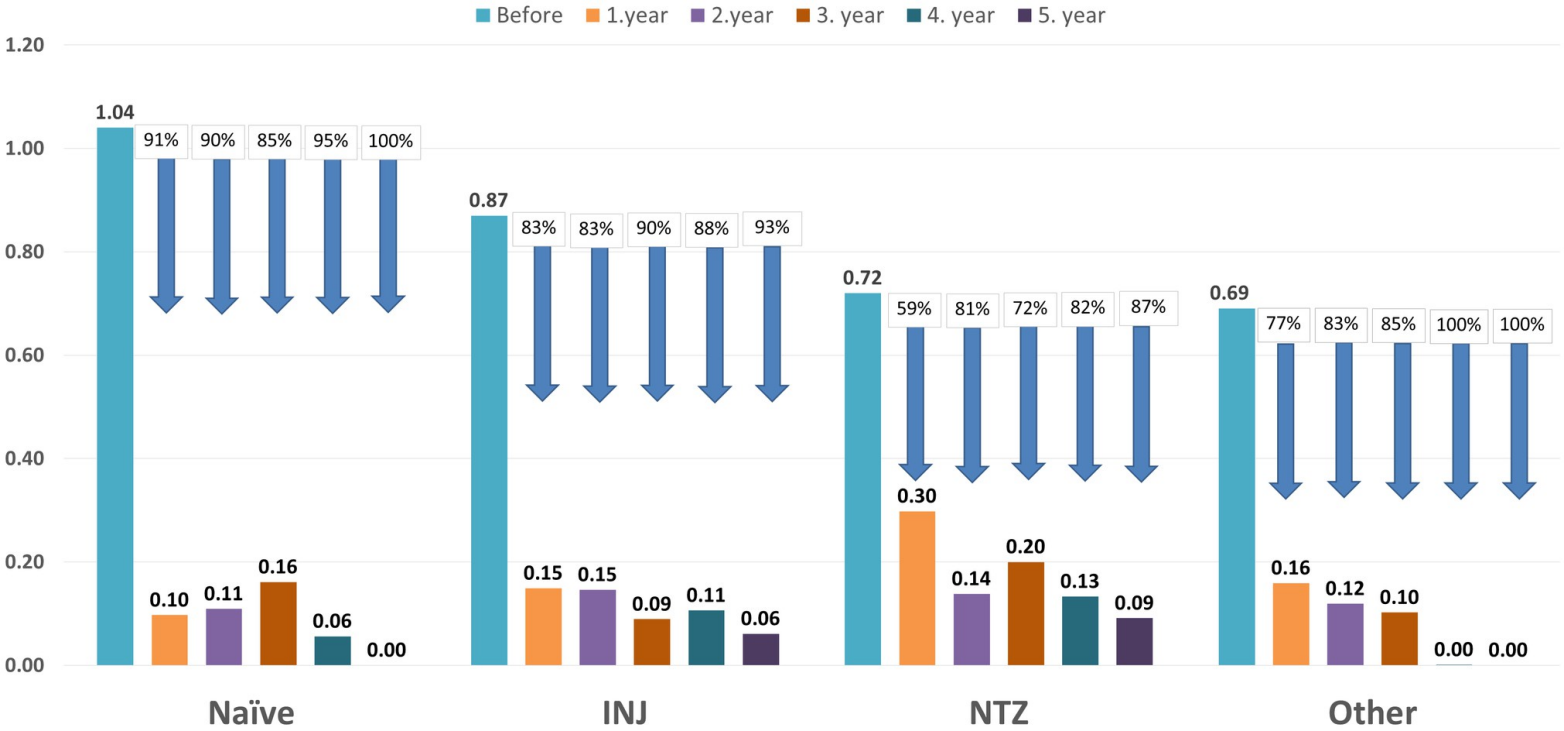
Annualised relapse rate of the study subgroups. Annualised relapse rate at baseline and 1, 2, 3, 4, and 5 years after initiation of treatment with fingolimod stratified by previous treatment.

The ARR for the whole cohort in the year before fingolimod treatment was 0.804 (95% CI: 0.74–0.86). A significant difference was seen regarding the ARR between the patient subgroups at baseline; the highest ARR was seen in the Naïve group (1.04, 95% CI: 0.68–1.41), followed by the INF (0.87, 95% CI: 0.77–0.97), NTZ (0.72, 95% CI: 0.65–0.80) and Other (0.69, 95% CI: 0.61–0.78) subpopulations. Treatment with fingolimod has proven to be highly efficacious already from the first year of treatment, which was sustained until study end across all subgroups. After one year of treatment, the ARR has significantly decreased to 0.185 (95% CI: 0.14–0.23, p<0.001). It has dropped from 0.835 (95% CI: 0.76–0.91), to 0.149 (95% CI: 0.11–0.19, p<0.001) after 2 years of treatment, from 0.822 (95% CI: 0.74–0.90), to 0.122 (95% CI: 0.08–0.17, p<0.001) after 3 years of treatment, from 0.882 (95% CI: 0.78–0.99), to 0.091 (95% CI: 0.04–0.14, p<0.001) after 4 years of treatment, and from 0.882 (95% CI: 0.75–1.01), to 0.097 (95% CI: 0.03–0.16, p<0.001) after 5 years of treatment (the variance seen in the baseline ARR is due to the different amount of patients in each year’s baseline population) (Fig 1, Table 3).

**Table 3:** Clinical effectiveness of fingolimod treatment.

	Proportion of patients free from relapse^a^	Cumulative proportion of patients free from relapse^b^	Proportion of patients free from 6 month CDP^c^	ARR^d^	Reduction in ARR compared tobaseline, %^e^
**Year 1**	**85.1%**	**85.1%**	**87.6%**	**0.185**	**77.0%**
**Year 2**	**88.1%**	**77.6%**	**81.9%**	**0.149**	**82.1%**
**Year 3**	**89.7%**	**73.8%**	**75.6%**	**0.122**	**85.2%**
**Year 4**	**91.6%**	**71.0%**	**68.2%**	**0.091**	**89.7%**
**Year 5**	**94.6%**	**69.6%**	**71.2%**	**0.097**	**89.0%**

**Notes**:

^a,b^: year 1, n = 570; year 2, n = 478; year 3, n = 370; year 4, n = 262; year 5, n = 184.

^c^: year 1, n = 450; year 2, n = 327; year 3, n = 242; year 4, n = 176; year 5, n = 111.

^d,e^: year 1, n = 509, baseline ARR = 0.804; year 2, n = 377, baseline ARR = 0.835; year 3, n = 286, baseline ARR = 0.822; year 4, n = 198, baseline ARR = 0.882; year 5, n = 124, baseline ARR = 0.822.

**Abbreviations**: ARR, annualised relapse rate; CDP, confirmed disability progression.

After one year of treatment, the ARR has decreased significantly compared to baseline and has constantly remained low thereafter, an effect which was also observed after stratification across all subgroups (p<0.001 for baseline vs the first year ARR for all subgroups, p<0.05 for each treatment year afterwards across all subgroups). Therapy naïve patients had the highest ARR at baseline and experienced the greatest reduction in the ARR after one year of treatment; however, from this point, no difference was seen in the ARR between the groups (p>0.05 for all comparisons) (Fig 1, Table 2). It is also noteworthy to mention that patients previously on natalizumab (whose disease is usually more severe and likely more challenging to keep in remission) experienced a 57% ARR reduction after one year of treatment, which remained consistently high and had elevated up to 87% in the later years (Fig 1).

**Fig 2:**
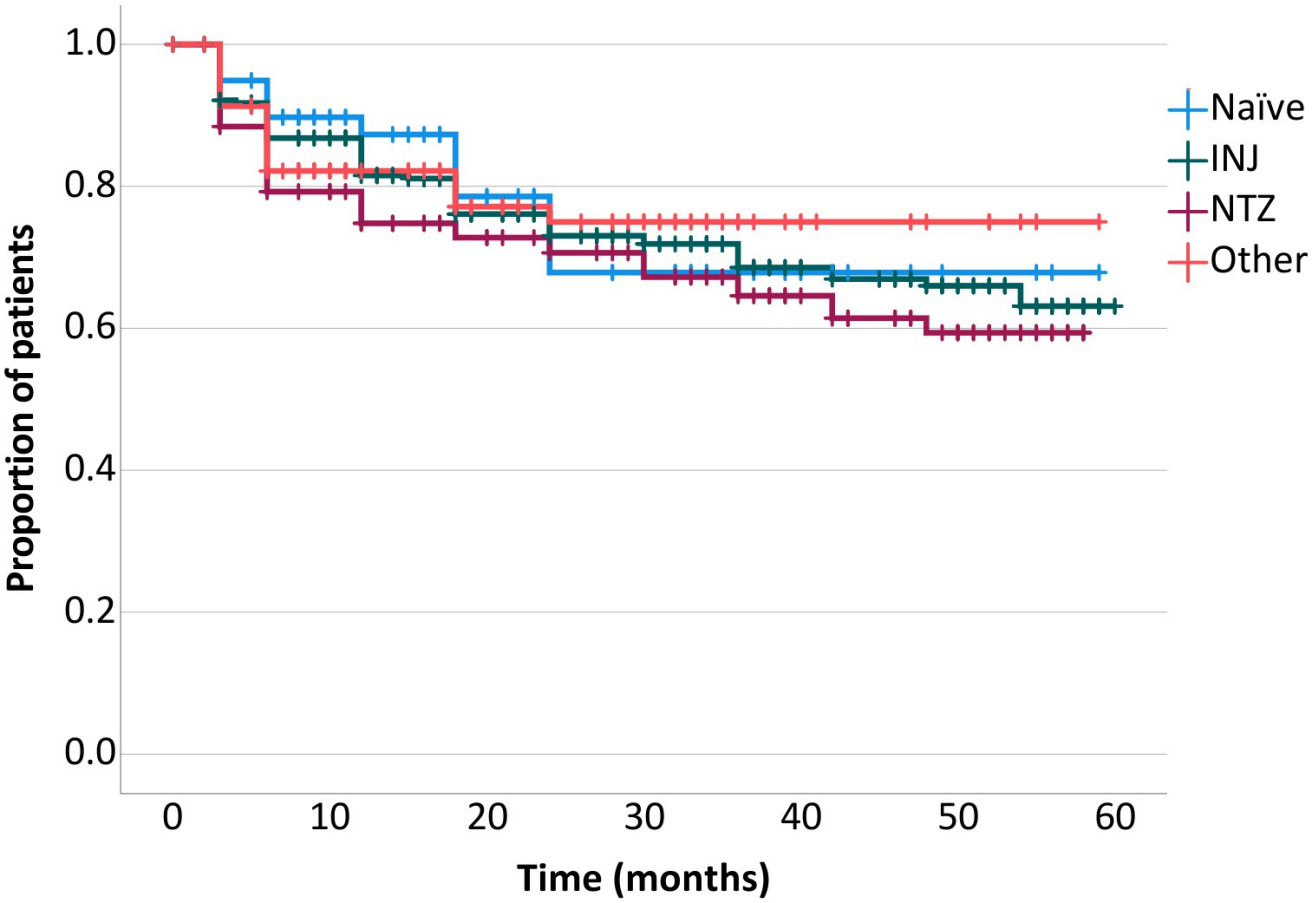
Kaplan-Meier estimates of the proportion of patients free of relapses.

When assessing the whole cohort, the cumulative proportion of relapse-free patients after 1, 2, 3, 4, and 5 years of treatment with fingolimod was 85.1%, 77.6%, 73.8%, 71.0%, and 69.6%, respectively. When stratified, no significant difference was seen in the ratio of relapse-free patients among the subgroups (79.5%, 79.0%, 71.0%, 65.6% of the patients remained relapse-free in the Other, Naïve, INJ and NTZ groups, respectively, log rank p = 0.588). In the 1^st^, 2^nd^, 3^rd^, 4^th^, and 5^th^ years 85.10%, 88.10%, 89.70%, 91.60%, and 94.60% of the patients did not experience a relapse (Fig 2, Table 2).

Regarding the EDSS score, compared to baseline (n = 520, mean: 2.67, SD: 1.524), a slight but continuous increase was observed, which did not reach statistical significance until the end of the third year. However, from this point onward, it has remained significant until study end (n = 114, mean EDSS: 3.32, SD: 1.924, p<0.001). After stratification significant difference was observed between the subgroups; patients in the Naïve group had the lowest, while patients in the NTZ group had the highest EDSS scores bot hat baseline and throughout the study (p<0.05 for each year’s comparison). Notably, compared to baseline, only patients in the Naïve group had lower EDSS scores at study end (Table 2).

**Fig 3:**
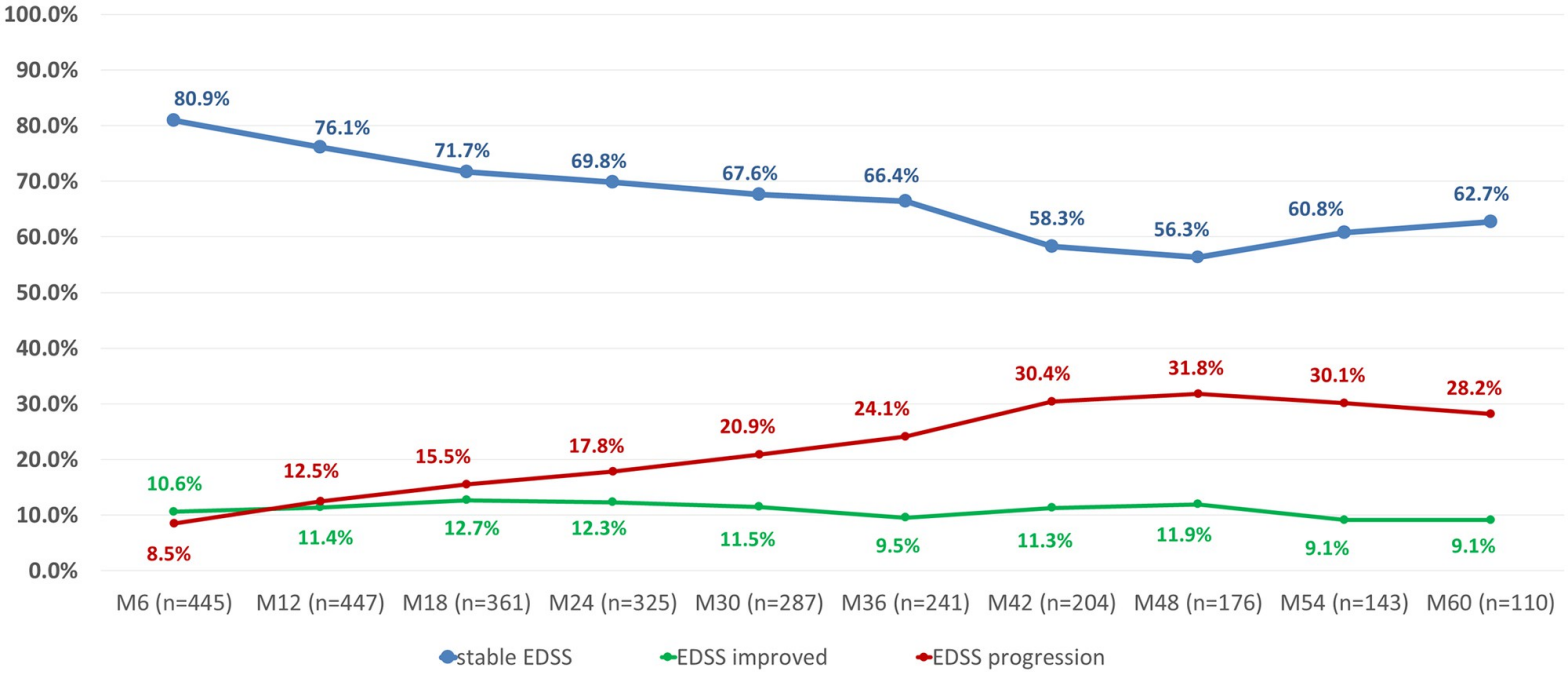
The proportion of patients with stable, improving and worsening EDSS scores.

**Fig 4:**
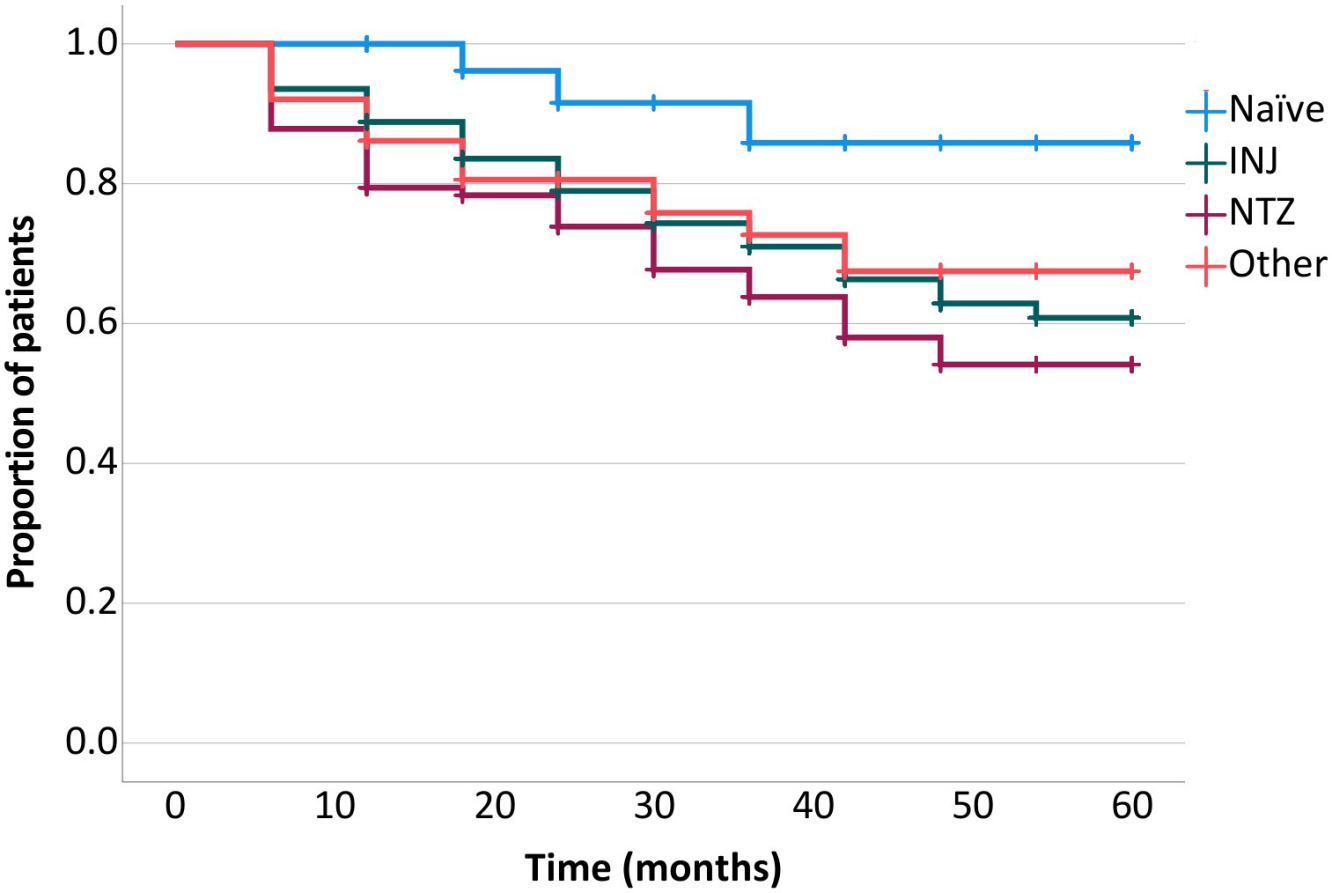
Kaplan-Meier estimates of time to proportion of patients with stable EDSS. **Note**: Log rank p = 0.033.

When examining the whole cohort, the proportion of patients with stable EDSS has fallen from 80.90% seen at the 6th-month visit to 62.30% observed at the 42^nd^-month visit, while the ratio of EDSS progressors has increased from 8.60% to 30.40% in the same time frame. However, from this point onward, the proportion of these patients remained relatively constant until study end. In contrast, the ratio of patients with improving EDSS scores has proven to be strikingly stable throughout the study; it ranged from 10.50% seen at study start to 9.00% measured at study closure (Fig 3). After subjects were stratified by EDSS stability, patients with improving EDSS scores proved to be the youngest (34.00±9.30, 39.94±9.38, 39.76±9.16 years of age at enrollment for patients with improving, stable and increasing EDSS scores, respectively, p = 0.01). Furthermore, a significant difference was observed between subgroups when patients with confirmed disease stability were stratified further based on previous treatment; the Naïve group had the highest, while the NTZ group had the lowest ratio of patients free of confirmed disability worsening (p = 0.033) at any point in the study (Fig 4). In contrast, there was no significant difference between the subgroups regarding the number of patients without a relapse during the study (p = 0.588). No other significant difference could be shown between these patients in any of the examined attributes (demographic parameters, previous treatment, ARR, p>0.05 for every comparison). The proportion of patients with an EDSS score lower than or equal to that seen before the study start was near, or over 70% at any time point during the observation period (Fig 3).

#### Quality of life

Compared to baseline after one year of treatment patients evaluated their HRQoL to be better in several dimensions; statistically significant increase was seen in the pain (p<0.023), energy (p = 0.018), health distress (p = 0.001), satisfaction with sexual function (p = 0.038) and change in health (p<0.001) subscales. No significant change was seen at year one or any point afterwards in the other subscales compared to baseline. After year one, no direct comparison could be made between a given year and baseline because of the lack of statistical power due to the fall in the number of questionnaires filled out by the patients (N = 148, 134, 51, 29, 13 at baseline, and after the 1st, 2nd, 3rd, and 4th year, respectively). However, even though statistical power was lacking, the same results between baseline and year one were observed as a tendency in each subsequent year.

Various clinical variables, which were deemed to have a potential influence on the different aspects of the patients’ HRQoL (EDSS score, previous treatment with natalizumab—indicative of a more aggressive disease, disease activity, presence of depression or anxiety, disease duration and time spent in the study) were evaluated. The most potent influencing factor was the change in EDSS score; it had a significant negative effect on 9 subscales (physical problems, role limitations due to physical problems, emotional well-being, energy, health perceptions, health distress, satisfaction with sexual functions and overall quality of life), followed by the presence of depression/anxiety as a co-morbidity, it influenced five subscales negatively (role limitations due to emotional problems, emotional well-being, social function, sexual function and overall quality of life subscale). Relapse-free patients evaluated their HRQoL to be better, they scored higher on every subscale, except for the sexual function and satisfaction with sexual function subscale. When patients were stratified based on their confirmed disease stability, EDSS progressors were found to have had scored worse on every scale influenced by the EDSS score. Also, patients with more follow-up time evaluated their HRQoL better on five subscales (pain, energy, health distress, satisfaction with sexual function, and change in health) (p<0.05 for every influencing factor and their respectively influenced subscale). At the 6^th^ month visit the CGI questionnaire was filled out for 532 patients (mean score 3.84), while at the 36^th^-month visit 261 questionnaires were completed (mean score 3.94). 143 patients scored the same, 46 patients scored lower. In comparison, 72 patients scored higher on the second evaluation, resulting in a significant difference between the two tests (p = 0.021), indicating a slight deterioration in the patients’ global state according to the treating physician’s opinion. The observed decline could not be attributed solely to the slightly deteriorating physical condition of the cohort, as no difference could be demonstrated between the CGI scores of patients with stable-, improving-, or worsening EDSS scores (χ^2^ = 0.478).

### Safety

#### First dose monitoring and cardiovascular safety

Consultation with a cardiologist specialist was necessary with 30 patients before commencing fingolimod treatment, for various reasons, mainly due to an already known cardiac disease. At the first dose monitoring visit and ECG was obtained from 548 patients, pathological findings were seen in 6 cases (1–1 case of 1st degree AV block, incomplete right bundle branch block, complete bundle branch block, sinus arrhythmia, and 2 cases of sinus bradycardia). The first dose of fingolimod had a clinically insignificant but statistically significant effect on the patients’ systolic and diastolic blood pressure and heart rate. Blood pressure was 122/77 Hgmm before the first intake vs 119/77 Hgmm after the 6 hour observation period (p<0.001), concomitantly the mean heart rate fell from 77/min to 67/min (p = 0.001). Prolonged observation was necessary in 28 instances. In one case, extended observation was required due to a temporary QT interval prolongation, while transient sinus bradycardia was the reason for the rest of the cases. Even though it was of no clinical relevance, a statistically significant decrease of heart rate and elevation in diastolic blood pressure was seen when comparing baseline and last visit values (79 vs 77 bpm, 78 Hgmm vs 81 Hgmm, p = 0.006 and p<0.001, respectively), meanwhile systolic blood pressure has shown no change (122 Hgmm vs 123 Hgmm, p = 0.057). Throughout the observation period 13 (1.80%) patients reported chest pain/discomfort, one myocardial infarction occurred, which was deemed unrelated to fingolimod treatment by the treating physician (Table 4). No patients with clinically significant cardiac adverse events nor rhythm abnormalities requiring medical intervention were identified during the study.

**Table 4:** Adverse events and serious adverse events of special interest during the follow-up period.

After first dose monitoring, n (%)^a^	First degree atrioventricular block	1 (0.2)
	Sinus arrythmia	1 (0.2)
	Incomplete right bundle branch block	1 (0.2)
	Right bindle branch block	1 (0.2)
	Sinus bradycardia	2 (0.4)
After continous fingolimod therapy, n (%)^b^		
Patients with any adverse event, n (%)		472 (65.56)
Patients with SAEs, n (%)		90 (12.50)
Total number of AEs		1324
Total number of SAEs		222
Adverse / **Serious adverse** events, n (%)	Lymphopenia	58 (8.0) /**2 (0.3)**
	Leukopenia	29 (4.0) /**3 (0.4)**
	Bradycardia	9 (1.3) /**1 (0.1)**
	**Acute myocardial infarction**	**1 (0.1)**
	Macular oedema	0 (0)
	Diarrhoea	17 (2.4)
	Pyrexia	32 (4.4) **/2 (0.3)**
	Nasopharyngitis	24 (3.3)
	Herpes infection (other than zoster)	13 (1.8)
	Herpes zoster	9 (1.3) /3 **(0.4)**
	Upper respiratory tract infection	64 (8.9)/**1 (0.1)**
	Urinary tract infection	14 (1.9)
	Pneumonia	4 (0.6) **/1 (0.1)**
	Abnormal liver enzymes	76 (10.6) /**10 (1.4)**
	Dizziness	29 (4.0)
	Headache	34 (4.7)
	Alopecia	14 (1.9)
	Hypertension	33 (4.6)
	Atrioventricular block	2 (0.3)/**2 (0.3)**
	Cardiac discomfort	1 (0.1)
	Chest discomfort/pain	13 (1.8) /**1 (0.1)**
	Cholelithiasis/cholecystitis	4 (0.6) /**1 (0.1)**
	Hepatic lesion	1 (0.1)
	Hypersensitivity	2 (0.3)
	Atrial fibrillation	2 (0.2)
	Abdominal pain/discomfort	15 (2.0) /**1 (0.1)**
	Infections (cumulative)	145 (20.1) /**8 (1.1)**
	**Progressive multifocal leukoencephalopathy**	**1 (0.1)**
Neoplasms, n (%)	Benign/**malignant** neoplasms	12 (1.7) /**7 (1)**
	**Lung adenocarcinoma**	**1 (0.1)**
	**Malignant melanoma**	**1 (0.1)**
	**Squamous cell carcinoma**	**1 (0.1)**
	**Breast cancer**	**1 (0.1)**
	**Bladder transitional cell carcinoma**	**1 (0.1)**
	**Cholangiocarcinoma**	**1 (0.1)**
	**Anogenital warts**	**1 (0.1)**

**Notes**: all data marked in bold represent the SAEs. AEs reported by a patient multiple times were still counted only once.

^a^: 100% = ITT, 570 patients;

^b^: 100% = SP, 720 patients.

**Abbreviations**: AE, adverse event; SAE, serious adverse event; PML, progressive multifocal leukoencephalopathy.

#### Adverse events, serious adverse events, and malignancies

During follow-up, 472 patients (65.56%) had experienced at least one adverse event; in total, 1324 AEs have been documented. Mainly infections (145, 20.13%; mostly urinary tract [14, 1.94%] and upper respiratory tract infections [64, 8.88%]), liver enzyme elevations (76, 10.55%), lymphopenia (58, 8.05%) and leukopenia (29, 4.02%), gastrointestinal and chest discomfort (15, and 13 cases, 2.08% and 1.80%, respectively), dizziness (29, 4.02%) and headache (34, 4.72%) have been reported. Long-standing bradycardia was reported 9 times (1.25%), herpes zoster infection occurred 9 times (1.25%). In our cohort, no patients were identified with macular oedema. Reasonable causality with fingolimod was assumed in 218 (20.6%) reported adverse events. The vast majority of events were resolved; deterioration was observed only in the case of 2 patients.

PML occurred in one patient (Table 4). The patient with PML had received natalizumab for 24 months before fingolimod, she had been started on natalizumab despite a positive JCV titer of 1.6. The reason for switching to fingolimod was the increasing JCV titer (to 3.6) and high risk for PML. The patient had a relapse (cognitive symptoms, gait ataxia, hemiparesis) just three weeks after fingolimod was initiated, a repeat MRI examination confirmed PML. Symptoms began to resolve after 2 weeks of antiviral, antibiotic and steroid treatment augmented with plasma exchange. Follow-up MRI scans (at months 1, 3, and 6) have proved the radiological resolution of PML as well, the patient recovered without any residual symptoms. No causality with fingolimod was assumed based on the patient’s previous medical history.

A total of 222 serious adverse events (SAE) have been reported by 90 patients (12.50%), of which 45 (20.27%) were severe relapses of the primary disease; the rest were of various origin (for the most common SAEs, see Table 4). In 41 cases, causality with fingolimod was presumed by the treating physician. The state of the patients has either stabilised, improved significantly, or even complete recovery has occurred in most of the SAEs (194, 87.38%). Deterioration or recovery with residual symptoms was seen in only a fraction of the cases (17, 7.65%); the fate of the remaining events was left unreported. Four patients have deceased during the observation period; no causality was assumed with fingolimod in either of the events. The causes of death were breast cancer, septicemia with concomitant paralytic ileus secondary to cholangiocarcinoma, status epilepticus with subsequent cardiorespiratory failure. At the same time, the last patient succumbed to the consequences of long-term bedriddance.

Fourteen participants have been diagnosed with a tumour, 9 patients had benign masses, 1 hemangioma, 2 lipomas, 1 thyroid adenoma, 1 breast adenoma, 1 unspecified benign tumour, and 3 skin papillomas were reported. Five malignant neoplasms have been detected; 1 malignant melanoma, 1 breast cancer, 1 cholangiocarcinoma, 1 bladder transitional cell carcinoma, and 1 anogenital wart. Our findings are within the range of expected malignancies in the general and MS populations. Our data is also in line with a most recent, WHO dataset based pharmacovigilance cohort study [36], which report noted a slightly increased cancer incidence with the concomitant use of various DMTs, of which fingolimod posed the smallest risk [36].

For the whole duration of the study, fingolimod was well-tolerated, with a high persistence lasting up to 60 months. The persistence with fingolimod after 60 months was 73.4%, which did not differ significantly between the subgroups (74.6% in the Naïve, 75.1% in the INJ, 63.9% in the NTZ, and 78.6% in the Other subpopulation (log rank p = 0.151). In total, 235 patients have terminated the study early, the leading cause of permanent discontinuation was the lack of effectiveness (74 cases, 31.48%) and AEs (mainly liver enzyme elevations, and other, unspecified side effects, 27–11.48% and 38 patients– 16.17%, respectively) (Table 5).

**Table 5:** Reasons for early discontinuation.

Cause	Patient No.	
	N	%^a^
**Withdrew consent**	**7**	**0,97%**
**Lost to follow-up**	**23**	**3,19%**
**Administrative**	**1**	**0,14%**
**Pathological laboratory findings**	**27**	**3,75%**
**Therapeutic insufficiency**	**74**	**10,28%**
**Side effect**	**38**	**5,28%**
**Other, unspecified**	**65**	**9,03%**

**Notes**: patients who did not complete 12 months follow-up but were active participants at study end were not considered early discontinuators. The total amount of dropouts was 237,

^a^: compared to the 720 patients comprising the safety population.

## Discussion

To date, even though the number of published or ongoing studies is rising, there is still only a limited amount of research dedicated to the evaluation of fingolimod’s effectiveness and safety in large real-world cohorts of patients. Studies assessing the demographic and clinical profile, quality of life, and disease activity of patients receiving fingolimod at the same time in a real-life clinical setting are even scarcer. Furthermore, apart from a select few [32, 37, 38], most of the studies examining fingolimod in a real-world practice published so far are heterogeneous in terms of the methodologies and/or the cohorts used. Some of them were conducted solely in a single centre [30, 39–43], some of them were posthoc analyses of the pivotal studies [44–46], some used a relatively small cohort [30, 39, 40, 43, 47, 48]. Meanwhile, others have focused only on a single objective [49–50]; except a few studies [51, 52], the follow-up period was surprisingly brief [43, 53–55] in several reports. Furthermore, some studies employed patients with both progressive and relapsing-remitting disease type [37]. Most of our findings corroborate the outcomes of the biggest real-life studies conducted with fingolimod so far [37, 38–51, 56].

Although several papers have been published evaluating the real-world effectiveness of fingolimod, most report outcomes are capped at 2 or 3 years. Studies are emerging with longer follow-up time, however [52]. The most informative, recently published studies supplying real-world information about the efficacy of fingolimod with at least 3 years of treatment come from Poland [32], Spain [37], Germany [57] and Turkey [52]. In our cohort, fingolimod was capable of reducing the incidence of relapses by up to 90% already from after the first year of treatment. This effect was sustained until study end. On the one hand, compared to the results from the studies mentioned above (always to the last available follow-up year the given study), we report a moderately (53–64%) lower ARR among our patients. On the other hand, a slightly lower proportion of our patients has remained progression (5%-20%) and relapse-free (9%-19%). An interesting finding, previously not reported, was that the most robust relapse rate reduction and the highest proportion of patients with a stable EDSS score were seen among the naïve patients, who also were the youngest patients of our cohort with the shortest disease duration. A plausible explanation for such compelling reduction in relapse rate in these patients might be explained at least, in part by their higher baseline ARR. Furthermore, the observed disease stability may be due to the fact that these patients were at the beginning of their highly active disease when neuroinflammation is more prominent than neurodegeneration, in which phase DMTs can exert their full potential in halting the disease. Considering that accumulation of long-term disability is associated with higher disease activity early in the course of MS, early initiation with high-efficacy DMTs is crucial [58, 59], it could lead to more favourable long-term outcomes [46]. Furthermore, ~2/3^rd^ (71.8%) of our cohort had a stable or improving EDSS score at study closure; EDSS progression was seen in only 28.2% of the patients.

The regular measurement of factors that negatively affect the HRQoL of MS patients is gaining ground in everyday clinical practice across the world. Furthermore, the evaluation of HRQoL representing the patients’ perspective is also an emerging endpoint in studies conducted in MS. The treating physicians’ opinion (expressed by the CGI scores) evaluated the patients’ overall status to slightly deteriorate over time. In contrast, the patients felt their condition improved in several aspects. In line with findings in the literature, the strongest determinant of the self-reported HRQoL was the physical state measured by the EDSS score. Yet, several other factors also significantly influenced different aspects of the patients’ quality of life. The fact that there was no difference between the EDSS score of the groups with worsening and stagnating HRQoL highlights the importance that the concept of HRQoL is far more complex and points beyond solely the physical state of the patients. This underlines the opinion that other aspects influencing the quality of life of MS patients Field (56) should be regularly assessed, further emphasising the need for complex, personalised care of these patients requiring teamwork from specialists of other fields of medicine. Even though our results are in line with other studies measuring the change in the HRQoL of MS patients, careful conclusions should be drawn from our findings due to the low number of available samples, especially after the first year. It is crucial to highlight, however, that as short as one year of treatment with fingolimod has had such a significant impact on the patients’ HRQoL that they ceased to be so concerned about multiple sclerosis’ impact their health (measured by the health distress and change in health subscales) as they were worried about it before treatment with fingolimod.

The frequency of all reported AEs and SAEs in our study was consistent with fingolimod’s SmPC and in concordance with other real-life studies examing a cohort of similar (or even bigger) size to ours [30], [37, 56, 57, 60]. Similarly to other real-world studies, long-term follow up in our cohort did not reveal any previously unreported safety signals [56, 57, 60–62]. Two open-label studies (START [63] and FIRST [50]), which dealt solely with the cardiovascular side effects of fingolimod upon the first-dose intake, have concluded that bradycardia is a transient, mostly asymptomatic event, also reported that AV-conduction abnormalities are infrequent and recover spontaneously. We report similar findings. The bradycardia experienced after the first-dose intake was a transient effect and no rhythm abnormalities necessitated intervention during the follow-up period in our cohort. Furthermore, even though the observed changes in systolic and diastolic mean blood pressure were statistically significant, they were clinically meaningless and comparable to that seen in other real-life studies.

Overall, roughly 2/3^rd^ of the patients reported at least one AE, while 12.5% experienced at least one SAE. The most frequently affected system organ classes were infections (upper respiratory tract and urinary tract infections), blood and lymphatic disorders (leukopenia and lymphocytopenia), liver enzyme elevations and nervous system disorders (headache and dizziness). There were four deaths during follow-up; the causes were heterogeneous, none of them attributable to fingolimod treatment.

The risk of an increased incidence of malignancies is one of the most feared and severe potential side effects of any drug with immunosuppressant properties. Even though prolonged fingolimod treatment has been linked to the increased incidence of basal-cell carcinomas [64], not one case was identified in our cohort. Additionally, we did not experience a rate of malignancies outside the range that is to be expected in the general and MS populations. Furthermore, the number of neoplasms reported in our study was similar to the rates of neoplasms documented in other long-term studies [56, 57, 60–62].

Our study was not without limitations. One major pitfall is the absence of radiological measures. Due to different imaging protocols, different scanner types and setups at the sites, direct comparison of the acquired images was unfeasible. A second shortcoming is the progressive shrinking of the study population; as enrollment did not stop 5 years before study end, there is a substantial number of patients with less than 5 years of follow-up. This may potentially bias results based on data gathered from later years. Similarly, the relatively low sample size in the subgroup analysis of the HRQoL questionnaires also warrants careful interpretation of the results. Nonetheless, our results support the long-term safety and efficacy of fingolimod in the treatment of RRMS.

## Conclusion

The subgroup analyses of patients based on previous therapy showed differential demographic and clinical characteristics at baseline additionally unearthed different clinical outcomes following treatment with fingolimod. Significant benefit was seen from fingolimod treatment in the whole cohort and across all evaluated subgroups, irrespective of their previously used DMTs. However, fingolimod’s most robust disease halting power was seen in the therapy naïve patients; even though previously treatment naïve patients had the highest disease activity at baseline, they experienced the most significant reduction in relapse rate. Furthermore, they showed improved long-term clinical outcomes (longer relapse and progression-free survival) compared to patients who switched from another DMT, especially from natalizumab. This further emphasises the importance of an adequate and timely treatment start [65, 66] with a DMT tailored to the patients’ needs and disease activity. The analysis of adverse events reported by patients using fingolimod in a real-world setting confirmed a long-lasting, favourable profile of both efficacy and safety, which spanned from study start to study end. Our results support the continuously increasing amount of evidence that confirms fingolimod’s long-lasting effectiveness and positive benefit/risk balance as a first- or second-line treatment in the treatment of RRMS patients in a real-life setting. Furthermore, this is the second biggest nationwide study (after PANGAEA and PANGAEA II) supplying real-world data on the efficacy safety of fingolimod and its impact on the patients’ quality of life for a follow-up period of up to 5 years.

## Supporting information

S1 Table Detailed study follow-up protocol. (DOCX)

S2 Table Complete list of study sites and respective number of patients enrolled in the study. (XLSX)
