# Supplementary material for: The safety and efficacy of fingolimod: Real-world data from a long-term, non-interventional study on the treatment of RRMS patients spanning up to 5 years from Hungary
Source: PLoS One. 2022 Apr 22;17(4):e0267346. doi: 10.1371/journal.pone.0267346 (PMC9032373; doi:10.1371/journal.pone.0267346)
Supplement: S1 Table — (DOCX) [file pone.0267346.s001.docx]

**Supplementary Table 1.:** Detailed study follow-up protocol

| **Assessed parameters** | **Study start, re-start** | **Study months** | | | | | **Optional visit** | **Early discontinuation^1^** | **Closing visit** |
| --- | --- | --- | --- | --- | --- | --- | --- | --- | --- |
|  |  | **1** | **3** | **6** | **9** | **12, 18, 24, 30, 36, 42, 48, 54** |  |  |  |
| Informed consent | X |  |  |  |  |  |  |  |  |
| MS disease history | X |  |  |  |  |  |  |  |  |
| Previous MS therapy | X | X | X | X | X | X | X | X | X |
| MSQoL-54 questionnaire | X |  |  |  |  | X^7^ |  |  | X |
| Concomitant diseases and therapies | X | X | X | X | X | X | X | X | X |
| MS status paremeters^2^ | X |  |  | X |  | X |  | X | X |
| CGI questionnaire |  |  |  | X |  |  |  |  | X |
| Fingolimod first dose monitoring^3^ | X |  |  |  |  |  |  |  |  |
| Pregnancy test | X |  |  |  |  |  |  |  |  |
| Vital parameters | X | X | X | X | X | X | X | X | X |
| Vaccination status | X |  |  |  |  |  |  |  |  |
| Ophtalmological examination^4^ | X^5^ |  | X | As necessary | | | As necessary |  |  |
| Complete blood count and biochemical panel^6^ | X | As deemed necessary, with the exception of transaminase levels, which were assessed mandatorily at months 1,3,6,9, and 12 | | | | | As necessary | As necessary | X |
| Adverse events | X | X | X | X |  | X | X | X | X |
| Serious adverse events | X | X | X | X |  | X | X | X | X |

1. All patients who left the study early were recalled for a control examination 3 months after discontinuation. This examination was considered the patient’s closing visit, where reasons for dropping out were assessed additional to a regular control neurological examination.

2. MS status evaluation consisted of the assessment of the EDSS score, the occurrence of a relapse, complete neurological examination, and a follow-up MRI scan if deemed necessary (at least once yearly, first at study start, and at months 12, 24, 36, 48, 60).

3. First dose monitoring procedures were always conducted in accordance with fingolimod’s European SmPC.

4. Three months after commencing treatment a routine ophthalmological examination was always conducted. If a patient ever reported any ophthalmological symptoms an urgent consultation with an ophthalmologist was always scheduled

5. Before therapy initiation all patients with diabetes and/or uveitis in their medical history were referred to an ophthalmologist, also they were routinely followed by their treating ophthalmologist during the study.

6. The conduction of any additional laboratory tests was always entrusted to the treating neurologist.

7. The MSQoL-54 questionnaire was evaluated at study start and yearly thereafter.
